# Diabetes Mellitus Prevalence by Uveitis Etiology at a Japanese Tertiary Center

**DOI:** 10.3390/diagnostics16132047

**Published:** 2026-06-30

**Authors:** Kei Wakatsuki, Kinya Tsubota, Masaki Asakage, Chihiro Maehara, Keiko Maruo, Yoshihiko Usui

**Affiliations:** Department of Ophthalmology, Tokyo Medical University, 6-7-1 Nishishinjuku, Shinjuku-ku, Tokyo 160-0023, Japan

**Keywords:** diabetes mellitus, uveitis, infectious uveitis, Behçet disease, sarcoidosis, prevalence, national prevalence estimates

## Abstract

Diabetes mellitus (DM) may influence susceptibility to ocular infection and inflammatory phenotypes; however, disease-specific DM prevalence across uveitis etiologies has not been well characterized. We evaluated DM prevalence across major uveitis entities in a large single-center cohort and compared observed prevalence estimates with age-specific national estimates from Japan. A total of 3163 adult patients out of 4751 patients newly diagnosed with uveitis at the Tokyo Medical University Hospital between 2004 and 2023 were included in the final analysis after excluding patients without DM-related laboratory parameters, those who declined to participate through the institutional opt-out procedure, pediatric patients, and patients with diabetic iritis. DM was defined as an HbA1c level of 6.5% or higher, current treatment for DM, or a random blood glucose level of 200 mg/dL or higher. For reference comparisons with national data, we analyzed seven adult entities with sufficient sample size. For each entity, expected DM prevalence was based on the corresponding national age-stratum estimate according to the mean age category of that entity. Observed and expected DM prevalence estimates were compared using Fisher’s exact test. Multivariable modified Poisson regression with robust variance estimation was used to estimate adjusted prevalence ratios (aPRs) for DM prevalence, including age, sex, and individual uveitis entities as covariates. Overall, 166 of 3163 patients (5.3%) had DM. DM prevalence differed substantially by etiology, ranging from 18.0% in endophthalmitis to 1.8% in Behçet disease. Selected infectious uveitis showed a higher crude prevalence of DM than selected noninfectious uveitis (10.4% vs. 3.8%; *p* < 0.01). In comparisons with corresponding national age-stratum estimates, the observed prevalence estimates of DM in endophthalmitis and herpetic iritis did not differ significantly from the expected national prevalence estimates, whereas acute retinal necrosis, intraocular lymphoma, sarcoidosis, and Behçet disease showed a significantly lower prevalence than expected. In the multivariable analysis, older age (aPR per 10-year increase, 1.49; 95% CI, 1.33–1.67; *p* < 0.001) and male sex (aPR, 2.11; 95% CI, 1.40–3.19; *p* < 0.001) were independently associated with DM prevalence, whereas individual uveitis entities were not significantly associated after adjustment for covariates. DM prevalence in uveitis is heterogeneous and disease-specific. Although selected infectious uveitis showed a higher crude prevalence of DM, comparisons with corresponding national age-stratum estimates suggested that some of the differences reflected the underlying age structure. Older age and male sex were the strongest independent correlates of DM prevalence.

## 1. Introduction

Diabetes mellitus (DM) is a common systemic disorder characterized not only by metabolic dysregulation but also by chronic low-grade inflammation, altered innate and adaptive immune responses, and impaired host defense [1,2]. These immunometabolic changes may increase susceptibility to infection, modify inflammatory responses, and influence ocular inflammatory manifestations [1,2]. In particular, DM is well recognized as a frequent comorbidity in infectious endophthalmitis and has been reported as a risk factor for ocular infection. Prior studies have also described DM among patients with uveitis; however, the extent to which DM prevalence differs across specific uveitis etiologies remains unclear, especially after accounting for age [3,4]. Because DM prevalence increases substantially with age in the general population, crude comparisons across disease entities may be difficult to interpret without considering age-specific reference data.

Although several reports have examined DM in selected uveitic conditions, comprehensive comparisons across major uveitis entities within a single tertiary referral cohort remain limited. A clearer understanding of disease-specific DM prevalence may improve interpretation of clinical background, refine etiologic assessment, and provide insight into the relationship between metabolic status and ocular inflammatory disease. In this study, we evaluated DM prevalence across major uveitis entities in a large single-center cohort and compared observed proportions with age-specific national prevalence estimates from Japan.

## 2. Methods

We retrospectively reviewed medical records of patients newly diagnosed with uveitis from 2004 through 2023. During the study period, 4751 patients were newly diagnosed with uveitis at our institution. Of these, 4251 underwent blood testing during the initial visit. Patients were excluded if DM-related parameters, including HbA1c and blood glucose, were not measured at the first visit (*n* = 691), if they declined to participate through the institutional opt-out procedure (*n* = 233), if they were pediatric patients (<20 years of age; *n* = 117), or if they had diabetic iritis (*n* = 47) (Supplementary Figure S1). Ultimately, 3163 adult patients were included in the final analysis. Patients with diabetic iritis were excluded because diabetic iritis is intrinsically associated with DM, and inclusion of this entity would introduce a circular association and artificially inflate the estimated prevalence of DM in the study cohort. In this study, DM was defined as HbA1c ≥ 6.5%, current treatment for DM, or a random blood glucose level ≥200 mg/dL at the initial visit. This definition was used as an operational definition for epidemiologic classification based on data available at presentation and was not intended to represent a definitive standalone clinical diagnosis of DM in every case. DM status was assessed using blood test data and medical record information obtained at the initial visit before the initiation of systemic corticosteroid therapy at our hospital. Uveitis etiology was assigned on the basis of the final diagnosis made by the treating uveitis specialists, according to the institutional diagnostic approach previously described [5].

For reference comparisons with national data, we focused on 7 adult entities with sufficient sample size: endophthalmitis, herpetic iritis, acute retinal necrosis (ARN), intraocular lymphoma, sarcoidosis, Vogt–Koyanagi–Harada disease (VKH), and Behçet disease. Age-specific national prevalence estimates were obtained from the 2023 National Health and Nutrition Survey of Japan, which reports the proportion of individuals “strongly suspected of having diabetes” by age group [6]. In that survey, individuals “strongly suspected of having diabetes” were defined as adults aged 20 years or older with HbA1c (NGSP) ≥ 6.5% or undergoing current treatment for diabetes. Thus, the definition used in the present study was not identical to that used in the national survey, because our operational definition additionally included patients with elevated random blood glucose at presentation. For reference comparisons, each uveitis entity was assigned to the corresponding national age stratum according to its mean age. Observed DM prevalence for each entity was compared with the expected prevalence derived from the corresponding national age-stratum estimate using Fisher’s exact test with the corresponding age-stratum-specific national reference counts, given the small number of DM cases in several subgroups. Comparisons between infectious and noninfectious categories were also performed using Fisher’s exact test.

To evaluate factors independently associated with DM prevalence, we performed multivariable analysis using a modified Poisson regression model with robust variance estimation and calculated adjusted prevalence ratios (aPRs) with 95% confidence intervals. The multivariable model was constructed using the disease-stratified analytic dataset with complete covariate data and included age (per 10-year increase), sex, endophthalmitis, herpetic iritis, intraocular lymphoma, sarcoidosis, Vogt–Koyanagi–Harada (VKH) disease, and Behçet disease as explanatory variables. Each disease entity was entered as an individual binary covariate. All *p* values were 2-sided, and *p* < 0.05 was considered statistically significant.

## 3. Results

**Figure 1:**
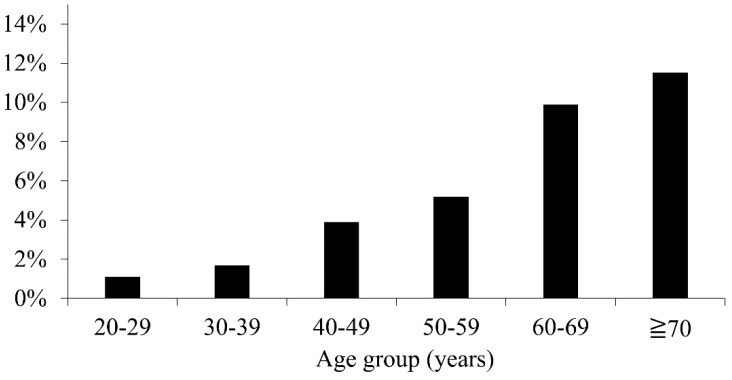
Prevalence of diabetes mellitus by age group among patients with uveitis. Bars indicate the prevalence of diabetes mellitus (DM) in each age group in the study cohort. DM prevalence increases with advancing age, showing a pattern broadly consistent with age-related trends in the general population. The age groups are 20–29, 30–39, 40–49, 50–59, 60–69, and 70 years or older.

**Figure 2:**
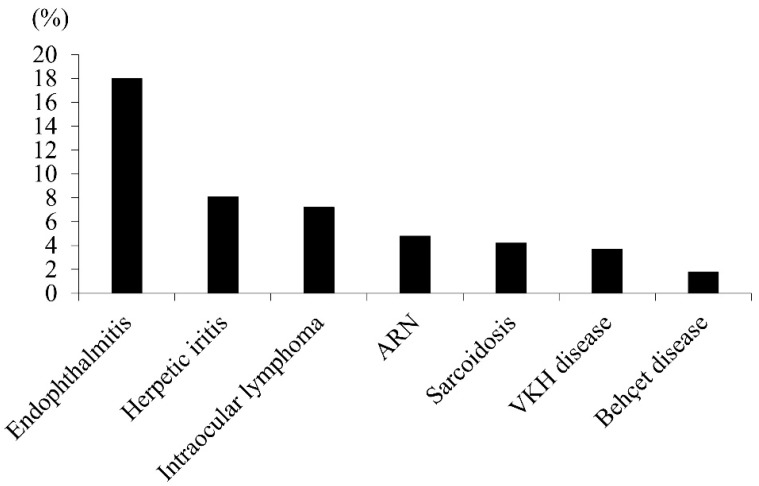
Prevalence of diabetes mellitus across selected uveitis entities. Bars indicate the prevalence of diabetes mellitus (DM) in selected uveitis entities. DM prevalence is highest in endophthalmitis and lowest in Behçet disease.

Of the 3163 adult patients included in the final analysis, 166 had DM, corresponding to an overall prevalence of 5.3%. Among patients with DM, 111 (66.9%) were men and the mean age was 62.6 ± 15.2 years. Among the 117 pediatric patients excluded from the primary adult analysis, DM was identified in one patient. Because comparable nationwide pediatric reference data were not available, pediatric cases were described only briefly and were not included in the primary comparative analysis. DM prevalence increased with age in the study cohort, broadly paralleling the age gradient observed in the national survey data (Figure 1). The disease-specific DM prevalence estimates for selected uveitis entities are shown in Figure 2. The highest prevalence was observed for endophthalmitis (25/139, 18.0%), followed by herpetic iritis (16/198, 8.1%) and intraocular lymphoma (10/139, 7.2%). Lower prevalences were observed for acute retinal necrosis (ARN; 5/105, 4.8%), sarcoidosis (12/286, 4.2%), Vogt–Koyanagi–Harada (VKH) disease (14/384, 3.7%), and Behçet disease (5/275, 1.8%) (Figure 2).

When grouped by etiology, selected infectious uveitis showed a significantly higher prevalence of DM than selected noninfectious uveitis (46/442 [10.4%] vs. 41/1084 [3.8%]; *p* < 0.01). However, comparisons with corresponding national age-stratum estimates suggested that crude differences were partly influenced by age distribution, as shown in Table 1.

The DM prevalence estimates in endophthalmitis and herpetic iritis did not differ significantly from the corresponding national age-stratum estimates based on the 2023 National Health and Nutrition Survey of Japan. In contrast, ARN, intraocular lymphoma, sarcoidosis, and Behçet disease showed a significantly lower DM prevalence than expected, whereas VKH disease showed a lower prevalence but did not reach statistical significance. These comparisons were performed using Fisher’s exact test with corresponding age-stratum-specific national reference counts because the number of DM cases was small in several subgroups. Prior systemic corticosteroid exposure before the initial visit was uncommon. The chart review identified only two patients with VKH disease who had received systemic corticosteroid therapy before presentation, and neither met the study definition of DM at the initial visit.

In the multivariable analysis, older age and male sex were independently associated with DM prevalence. The adjusted prevalence ratio (aPR) for DM was 1.49 (95% CI, 1.33–1.67; *p* < 0.001) per 10-year increase in age and 2.11 (95% CI, 1.40–3.19; *p* < 0.001) for male sex. Endophthalmitis (aPR, 1.56; 95% CI, 0.89–2.75; *p* = 0.12), herpetic iritis (aPR, 1.61; 95% CI, 0.81–3.22; *p* = 0.176), and intraocular lymphoma (aPR, 1.29; 95% CI, 0.54–3.11; *p* = 0.571) showed positive but nonsignificant associations with DM prevalence. In contrast, sarcoidosis (aPR, 0.78; 95% CI, 0.34–1.75; *p* = 0.54), Vogt–Koyanagi–Harada disease (aPR, 0.49; 95% CI, 0.15–1.55; *p* = 0.224), and Behçet disease (aPR, 0.35; 95% CI, 0.05–2.49; *p* = 0.293) showed inverse but nonsignificant associations with DM prevalence. These findings indicate that age and sex were the strongest independent correlates of DM in this cohort.

## 4. Discussion

In this 20-year tertiary-center cohort, DM prevalence differed across uveitis etiologies, suggesting that the relationship between DM and uveitis may be disease-specific rather than uniform [3,7]. Although DM was more common in selected infectious uveitis entities than in selected noninfectious entities in crude analyses, comparisons with corresponding national age-stratum estimates suggested that some of these differences reflected the underlying age structure. Specifically, the observed DM prevalences in endophthalmitis and herpetic iritis were comparable to the expected prevalences based on age-specific national estimates. Because DM prevalence increases substantially with age in the general Japanese population, crude differences across uveitis entities may partly reflect differences in age distribution [8]. Conversely, several entities, including ARN, intraocular lymphoma, sarcoidosis, and Behçet disease, showed lower-than-expected DM prevalences. VKH disease also showed a lower prevalence of DM than expected, although this difference did not reach statistical significance. These findings suggest that DM prevalence among patients with uveitis is not uniform across disease entities and may vary according to disease-specific clinical background, referral patterns, demographic structure, and underlying inflammatory mechanisms.

Several explanations are plausible. DM-associated immune dysregulation may preferentially increase susceptibility to selected infections, whereas its relationship with immune-mediated uveitis appears less consistent [1,2]. Chronic low-grade inflammation, altered host defense, and metabolic dysfunction may influence ocular inflammatory phenotypes, but these effects may differ depending on the underlying uveitis etiology. Alternatively, referral patterns and diagnostic workups in tertiary uveitis practice may introduce selection or detection bias. Treatment-related factors and metabolic comorbidities may also modify glucose status and thereby influence the observed association between DM and specific uveitis entities. The lower-than-expected prevalence estimates observed in Behçet disease and several noninfectious entities are also of interest. These diseases may arise in patients with metabolic, immunologic, or immunogenetic backgrounds that differ from those typically associated with DM. For example, Behçet disease is influenced by HLA-related immunogenetic susceptibility [9,10]. However, genetic background was not evaluated in the present study. Therefore, this interpretation remains speculative, and the lower DM prevalence estimates observed in these entities should be interpreted cautiously.

Several limitations should be acknowledged. First, this was a single-center retrospective study conducted at a tertiary referral institution, and the case mix may not fully reflect the general Japanese population. Second, the study period was long, and although no major institutional changes in the basic policy for diabetes testing or referral were identified, subtle changes in laboratory practice, referral patterns, clinical awareness of DM, and the epidemiologic spectrum of uveitis may have occurred over time. Third, the definition of DM in this study was not identical to that used in the national survey. The present study used a slightly broader operational definition by additionally including patients with a random blood glucose level ≥200 mg/dL at presentation; therefore, the comparisons with national survey estimates should be interpreted as reference comparisons rather than strict comparisons based on an identical case definition. A strict clinical diagnosis of DM may require presentation of symptoms, repeat testing, and comprehensive evaluation by physicians specializing in diabetes care [11]. However, such confirmatory information was not available for all patients in this retrospective ophthalmology-based dataset. Fourth, the number of DM cases was small in several subgroups, including ARN, Behçet disease, and intraocular lymphoma, limiting statistical power and precision. Therefore, the subgroup findings should be interpreted cautiously, and the absence of statistical significance should not be regarded as definitive evidence of no association. Fifth, detailed DM subtype information, including type 1 and type 2 DM, was not consistently available. Sixth, important metabolic and clinical confounders, including BMI, obesity, hypertension, smoking, prior treatment history, and medications other than corticosteroids, could not be comprehensively assessed. Finally, pediatric patients were excluded from the primary adult analysis. Although DM was identified in one of the excluded pediatric patients, interpretation was limited because comparable nationwide pediatric reference data were not available.

In conclusion, DM prevalence in uveitis is heterogeneous and varies according to disease entity. Although selected infectious uveitis showed a higher crude prevalence of DM than selected noninfectious uveitis in this study, comparisons with corresponding national age-stratum estimates suggested that some of these differences were influenced by the underlying age structure. Older age and male sex were the strongest independent correlates of DM prevalence, whereas individual uveitis entities were not significantly associated after multivariable adjustment. These findings indicate that the burden of DM in uveitis should be interpreted in the context of both demographic characteristics and disease-specific clinical background.

## Figures and Tables

**Table 1:** Diabetes mellitus prevalence in selected uveitis entities.

Category	Entity	Mean Age, y	DM Cases/Total (%)	Expected DM Prevalence, %	*p* Value
Infectious	Endophthalmitis	66.3	25/139 (18.0)	17.3	0.89
Infectious	Herpetic iritis	57.6	16/198 (8.1)	13.5	0.06
Infectious	Acute retinal necrosis	57.5	5/105 (4.8)	13.5	0.01
	Subtotal infectious		46/442 (10.4)		
Noninfectious	Intraocular lymphoma	63.9	10/139 (7.2)	17.3	<0.01
Noninfectious	Sarcoidosis	51.9	12/286 (4.2)	13.5	<0.01
Noninfectious	VKH disease	46.5	14/384 (3.7)	7.2	0.06
Noninfectious	Behçet disease	40.0	5/275 (1.8)	7.2	<0.01
	Subtotal noninfectious		41/1084 (3.8)		

VKH, Vogt–Koyanagi–Harada. Expected prevalence was derived from the corresponding national age-stratum DM prevalence estimate based on the mean age category of each entity from the 2023 National Health and Nutrition Survey of Japan. *p* values were calculated using Fisher’s exact test with the corresponding age-stratum-specific national reference counts.

## Data Availability

The data presented in this study are available from the corresponding author upon request. The data are not publicly available because of institutional and privacy restrictions.

## Supplementary Materials

The following supporting information can be downloaded at: https://www.mdpi.com/article/10.3390/diagnostics16132047/s1, Figure S1: Flowchart of patient selection. A total of 4751 patients were newly diagnosed with uveitis during the study period. Of those patients, 4251 underwent blood testing at the initial visit. After excluding patients without DM-related laboratory parameters, those who declined to participate through the institutional opt-out procedure, pediatric patients, and patients with diabetic iritis, 3163 adult patients were included in the final analysis. DM indicates diabetes mellitus.
