# Supplementary figures and images for: Diabetes Mellitus Prevalence by Uveitis Etiology at a Japanese Tertiary Center
Source: Diagnostics (Basel). 2026 Jun 30;16(13):2047. doi: 10.3390/diagnostics16132047 (PMC13359844; doi:10.3390/diagnostics16132047)

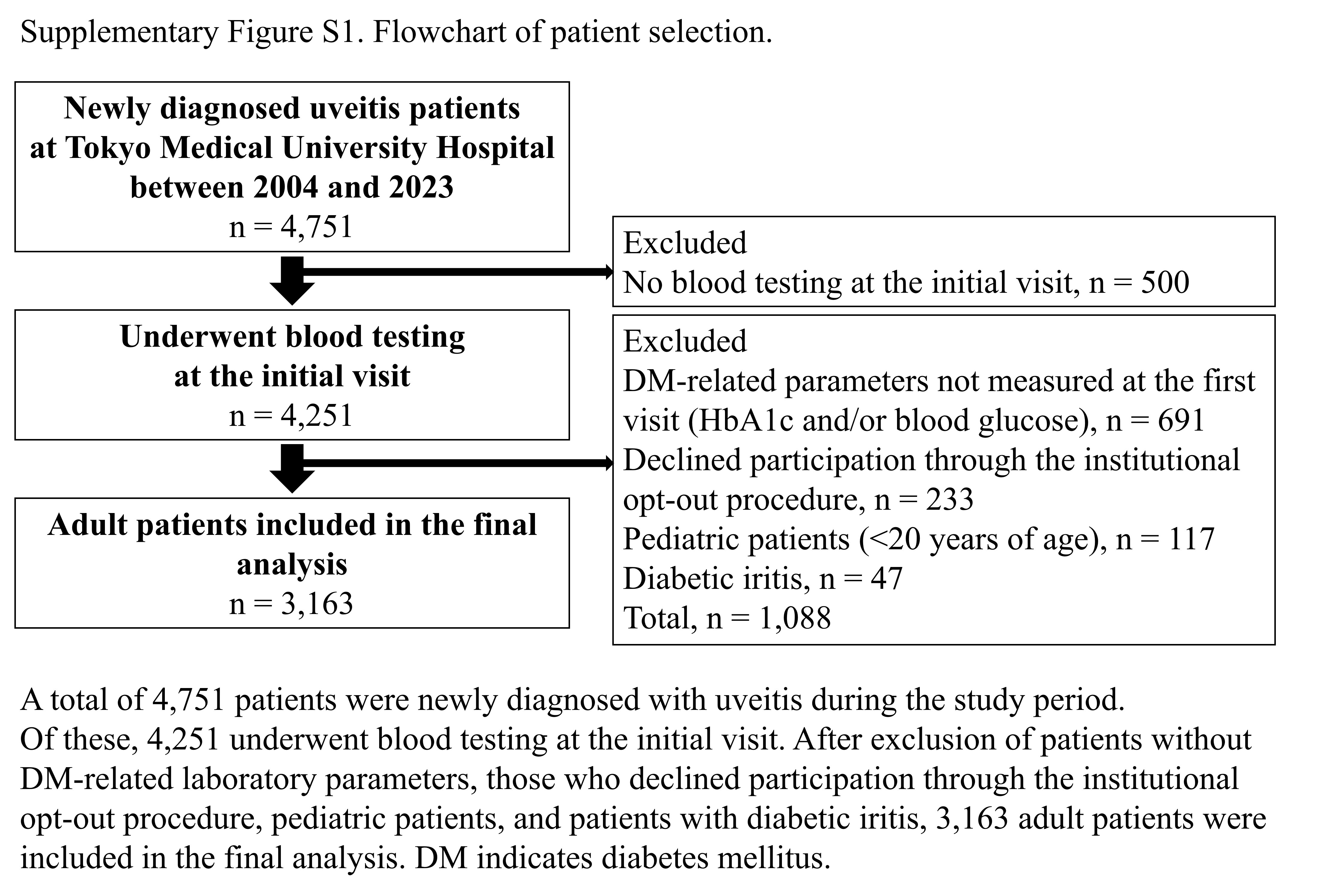

Supplement: Supplementary file 1 [file diagnostics-16-02047-s001.zip › diagnostics-4384404-supplementary.jpg]
